# Relating Competitive Golfers’ Perceived Emotions and Performance

**DOI:** 10.1177/00315125211005938

**Published:** 2021-04-12

**Authors:** Erik Lundkvist, Henrik Gustafsson, Gunilla Björklund, Paul Davis, Andreas Ivarsson

**Affiliations:** 1 Department of Psychology, Umeå University, Umeå, Sweden; 2 Department of Educational Studies, Karlstad University, Karlstad, Sweden; 3 Physical Activity and Health Unit, Swedish School of Sport and Health Sciences, Stockholm, Sweden; 4 Swedish National Road and Transport Research Institute (VTI), Umeå University, Umeå, Sweden; 5 Department of Psychology, Umeå University, Umeå, Sweden; 6 Centre of Research on Welfare, Health and Sport, Halmstad University, Halmstad, Sweden

**Keywords:** golf competition, negative affect, task-oriented coping, perceived control, performance under pressure

## Abstract

The present study examined relationships between golfers’ self-perceived emotions (e.g., irritability, nervousness, tension), task-oriented coping, perceived control, and performance during a golf competition. We implemented a process-oriented golf analysis in which competitors rated these variables hole-by-hole in a competitive golf round. Within a two-level Bayesian multivariate autoregressive model, we showed that (a) within persons, emotions and task-oriented coping were *reactions* that stemmed from performance on the previous hole; and (b) between persons, player *skill level* predicted both better scores and the ability to limit the influence of negative affect on performance. These findings highlight the complex nature of the relationship between emotions and performance. Future studies might use a similarly ecologically valid research design to more precisely measure aspects of time and potentially moderating effects of player skill level and personality. An increased understanding of the dynamic relationship between emotions and performance can promote the development of effective psychological interventions for optimal performance outcomes.

It is widely acknowledged that emotions in sport are highly influential for athletes’ performance and wellbeing (Amiot et al., 2004; Doron & Gaudreau, 2014; Gucciardi & Dimmock, 2008; Schantz & Conroy, 2009). Numerous theories and models (e.g., Hardy, 1996; Mullen & Hardy, 2000) have proposed that negative emotions (e.g., anxiety, anger), and perceived ability to handle competitive situations (e.g., perceived control, task-oriented coping) can hinder or facilitate athletes’ decision-making, concentration, and motivation to perform to one’s full ability (Allen et al., 2013; Robazza, Pellizzari, & Hanin, 2004; Woodman et al., 2009). Traditionally, applied sport psychology has promoted the view that athletes’ performance may be optimized through emotional regulation strategies aimed to induce individual zones of optimal functioning (Jones, 2003). Alternatively, theories based on the third wave of cognitive behavioral therapy (Hayes et al., 2004) suggest that accepting both positive and negative emotions may minimize performance disruption and athlete distress (Gardner & Moore, 2004, 2012).

Player emotions are of particular interest in golf because of temporal space between performance epochs that may elicit high within-person variation across time during an 18-hole round (Schantz & Conroy, 2009). Although past golf literature reviews (e.g. Hellström, 2009) have called for more study of the relationships between emotions and performance, investigations in this area have been scarce. Most studies have focused on golf putting within laboratory environments (e.g., Chamberlain & Hale, 2007; Woodman & Davis, 2008) or on the athlete’s pre-competition mood or emotions, omitting a focus on in-the-moment emotional states preceding the execution of a particular shot during competition (Hellström, 2009). Although, Schantz and Conroy (2009) studied the relationship between emotional states (e.g. negative affect) and performance during a golf practice round, no study, to our knowledge, has researched this relationship within a competitive round.

Emotions are usually perceived as short-term intense reactions to a specific occurrence, while moods are longer lasting with more diffuse causes (Mellalieu, 2003). Affects are often defined as emotional displays, although researchers have often measured the total affective state rather than separated emotional displays (Batson et al., 1992). Researchers’ reliance on latent constructs like affect has risked inattention to detailed specificity and a loss of explanatory power associated with certain discrete emotions (Kranzbüler et al., 2020; Lazarus, 2000). In a review of emotions induced by performance outcomes, Allen et al. (2011) observed that athletes who saw themselves as performing worse than their personal performance standard experienced increased anger and dejection. These associations were also evident in competitive sports studies in which poor performance predicted negative emotions (e.g., negative affect) and good performance predicted higher problem focused coping and perceived control (Doron & Gaudreau, 2014; Schantz & Conroy, 2009). Studies undertaken within laboratory settings in which test conditions offered greater variable control have shown variable player characteristic influences on the emotion-performance relationship (Balk et al., 2013; Gucciardi & Dimmock, 2008). Specifically, relatively lower skilled golfers (mean handicap of 14.76) performing in high-pressure conditions experienced a decline in performance accuracy compared with low pressure conditions, while relatively skilled golfers (mean handicap of 6.35) performed better in high-pressure competition than in practice conditions (Gucciardi & Dimmock, 2008). Additionally, golfers who experienced heightened arousal (indicated by increased heart rate and elevated self-reported state anxiety), performed worse than golfers who experienced lower arousal (Balk et al., 2013).

In attempts to optimize performance, sport psychology researchers have extensively studied athletes’ coping strategies (Nicholls & Polman, 2007). In particular, Higgins and Endler (1995) clustered coping strategies into three types: task-oriented, emotion-oriented, and avoidance-oriented. Task-oriented coping strategies are problem focused and include a clear intent to act directly on the task at hand in order to address the source of stress, regain control, and revaluate one’s thoughts. Emotion-oriented coping focuses on altering the emotional responses rather than task performance. Avoidance-oriented coping attempts to avoid emotionally arousing situations or deny the situation’s existence (Higgins & Endler, 1995; Lazarus & Folkman, 1984). In sport competitions, including golf, task-oriented coping has been found to be most predictive of a successful performance (Amiot et al., 2004; Gaudreau & Blondin, 2004; Nicholls et al., 2012).

Closely related to coping strategies and performance is perceived control (Skinner & Zimmer-Gembeck, 2011). Athletes who have a strong belief in their personal ability or control as opposed to either luck or their opponent’s ability have been shown to perform better than those who tend to attribute this control or ability to either luck or the opponent (Wood & Wilson, 2012). In sport psychology research, low perceived control has been associated with negative emotions, such as anxiety (Jordet et al., 2006) and to poorer performance outcomes (Schantz & Conroy 2009). Higher levels of perceived control have been associated with greater skill levels and better performance (Gucciardi & Dimmock, 2008).

Previous studies with complex research designs in domains beyond sport, have shown that emotions and performance have interchangeable reciprocal effects over time (Beal et al., 2005). Considering the dynamic nature of emotional responses within sport competitions (Cerin et al., 2000), it is important to study the reciprocal effects of performance and emotions during competitions of a longer duration. Since a golf competition can last up to five hours, there may be wide ranging emotional states and performance outcomes that vary with self-appraised perceived control across different stages of play.

Our aim was to perform a conceptual replication (Neuliep, & Crandall, 1993) of Doron and Gaudreau (2014)’s and Schantz and Conroy’s (2009) studies, using a point-by-point analyses of player perceptions during ecologically valid competitive play. Thus, during actual golf competitions, we examined whether golfers’ hole-to-hole self-perceptions of negative affect, perceived control, and task-oriented coping would predict their performance on the subsequent hole, and/or whether prior performance on a preceding hole would predict these post-hole self-perceptions. Based on previous related research (Doron & Gaudreau, 2014; Schantz & Conroy, 2009), we hypothesized that negative affect, perceived control, and task-oriented coping would minimally influence players’ performances on the upcoming hole. Further, we expected performance outcomes on the previous hole to guide golfers’ subsequent affect, perceived control, and task-oriented coping.

Since past researchers have found temporal differences in athletes’ emotional responses in relation to pre- middle- and post- competition time blocks (Cerin et al., 2000), we explored temporal aspects of emotional responses across golf competitions by dividing the competitive golf round (i.e., 18 holes) into thirds (i.e., 6-hole blocks). This enabled us to study whether the relationships between emotions and performance remained consistent through a competitive round or varied with the stage of the round (i.e., beginning, middle, end). Because some researchers have argued that discrete emotions (versus mood states) may provide a more detailed explanation of the relationship between emotions and performance (Lazarus, 2000) a second exploratory aim of the present study was to analyze emotion-performance relationships with three specific negative emotional states (i.e., nervousness, irritation, and tension) independently, rather than in terms of the broad latent construct of negative affect.

## Method

### Participants and Procedure

Participants were 28 golfers competing at levels ranging from successful elite to semi-elite (Swann et al., 2015) with a mean handicap of 4.94 (*SD* = 3.58, *MED* = 4.60, range = −2 to 12.2). The golfers’ mean age was 28.35 years (*SD* = 10.08, *MED* = 26, range = 17 to 55) and their average golf experience was 12.47 years (*SD* = 8.52, *MED* = 10, range = 1 to 35). This sample contained one female and 27 males. Data were collected at the three first scratch competitions of a Swedish regional tour consisting of six competitions. The three competitions were all played from the back tee, where the first two first courses had a scratch value of .7 (a scratch golfer is assumed to play the course .7 over par) and the final one had a scratch value of 1.4. The study was first presented on the golf tours Facebook page. Upon initial contact with players when they checked into the organizer’s desk at competition, one of the researchers explained the aim and procedure of the study. Approximately 50 percent of the contacted players agreed to participate in the study, although two persons used their right to withdraw from the study during the competition.

The players who agreed to participate were provided with a small booklet designed to fit unobtrusively in the players’ bags. The first page of the booklet outlined the study’s aims, followed by an informed consent form, and then a page collecting background information (i.e., age, years played, and handicap). That section was followed by 18 pages with questionnaire items designed to measure the three test constructs (negative affect, perceived control, and task-oriented coping), on a ten-centimeter visual analogue scale (VAS; Gould, Kelly et al., 2001). One of these pages was to be completed prior to playing each hole. In consideration of the complexity of some of the constructs measured, we provided an extensive definition of each at the beginning of the booklet and a more concise definition on each page. Upon completion of the round, participants returned the test booklets and were thanked for their involvement in the study. We provided them with a three pack of golf balls as a token of appreciation. Although we collected data during three competitions, we used only the golfer’s best round if golfers took part in more than one data collection.

### Measures

The test constructs *perceived control*, *negative affect*, and *task-oriented coping* were adapted from Doron and Gaudreau (2014), who made a point-by-point analysis of performance in a fencing tournament; we reframed these constructs to suit golfing purposes.

Perceived control was measured by a single item, “*the meaning of perceived control are the emotions you have regarding your ability to change or manage the situations during a golf competition. At this exact moment, how is your ability to change or manage the game situations?*” The question was answered on a VAS-scale using the anchors 1 = “*have no ability at all*” to 10 = “*definitely have the ability*”.

Negative affect was measured by calculating the golfer’s average score across three different questions covering three types of affective states (i.e., nervousness, irritability and tension) with a broad description in the outline: “*the meaning of affect relates to different emotions you can have during a golf competition, for example feeling easily irritated, nervous, or tense.*” The definition was then followed by: “At this moment, to what extent are you easily irritated?”; “At this moment, to what extent are you nervous?”; and “At this moment, to what extent are you tense?” The questions were each answered on a VAS-scale anchored with 1 = “*not at all*” to 10 = “*very much*”. A similar approach was applied to that of Doron and Gaudreau (2014).

**Task-oriented coping** was measured by first explaining the concept with the following statement on every page in the booklet: *“task-oriented coping means the strategies you use to handle the situations you face and to solve problems. For example, the ability to keep concentration high, search for relevant information, use advice from your coach, analyze the strategy you have on a hole or a certain shot, improve exertion to manage the whole round, find solutions to the problems you get on the course and find a plan for how to hit better shots.* This statement was followed by the question: “*At the moment, how are your abilities to handle the situations you are in or solve problems which you face on the course?*” This was answered on a VAS scale where 1 = “*have no possibilities*” and 10 = “*have very good possibilities*”.

**Score** was standardized from participants’ performance relative to the par score indicated for each of the holes. Specifically, par was coded as 0, birdie as −1, eagle as −2, bogey 1, double bogey as 2, triple bogey as 3 and following the same logic up to 6, which was the maximum score per hole.

**Skill level** was measured using the playing handicap (HCP) that the player possessed at the time of data collection. The lower the handicap a player possessed, the higher the player’s playing ability or skill level.

**Experience** was measured as the number of years a player had played golf.

### Statistical Analyses

We analyzed descriptive statistics using JASP statistics (Jasp Team, 2018). We specified two-level Bayesian multivariate autoregressive models, using M*plus* 8.1, to test whether negative affect, perceived control and task-oriented coping predicted the golfers’ performance on the following golf holes and/or whether their performances on a golf hole predicted their negative affect, perceived control, and task-oriented coping for the next hole. We divided each golf round into beginning, middle, and end (i.e., holes 1–6, holes 7–12 and holes 13–18). Within this type of model, it is possible to separate the within person relationships between these variables from the between person relationships (permitting comparisons of golfers of different skill levels (Schuurman et al., 2016). We chose Bayesian estimation as it is a robust way to test temporal relationships with auto-regressions and lagged effects in small participant samples (Hox et al., 2012; Muthén & Asparouhov, 2012). At level 1, the within person associations between negative affect, perceived control, task-oriented coping, and score (performance) were tested for two temporal possibilities – first, whether emotions experienced just before the first shot of the hole predicted the score on this hole, and, second, whether the score on a hole predicted the golfer’s emotions experienced after that hole and before the first shot on the upcoming hole. At level 2 we tested whether the within-person associations tested at level 1 were moderated by individual golfer differences such as skill level (HCP) and golfing experience (years played). The main analysis included models tested for each separate hole in the whole competitive round. We also explored whether the relationships between these variables differed across temporal aspects of the competition (i.e., the first six holes, the middle six holes, and the last six holes), since different emotions are known to have different effects over time. (For example, nervousness effects usually level out over time (Cerin et al., 2000)). We tested nested models using deviance information criteria (DIC) in which a lower value indicates a better data fit (Ando, 2007; Spiegelhalter et al., 2002). To test statistical credibility, we used the credibility interval (CI) for all parameters within the models. The credibility interval indicates, the probability (i.e., 95%) that the observed data for the parameter of interest lies between two values. We followed recommendations from Zyphur and Oswald (2015) such that statistical credibility was reached when the 95% CI did not include zero.

## Results

**Figure 1.**
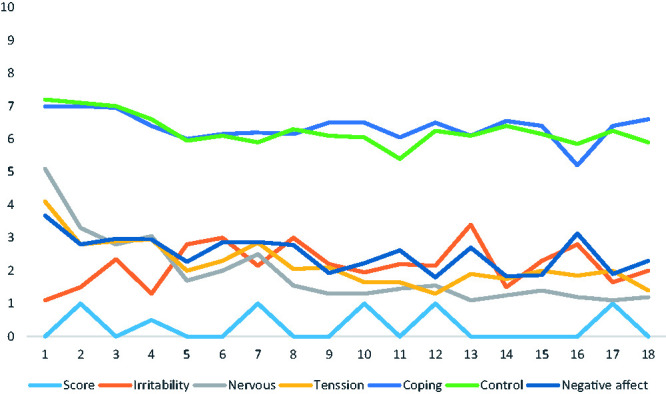
Median Self-Ratings for Participants’ (a) Irritability, Nervousness, Tension, Coping, Control and Negative Affect Measured Prior to Each Hole and (b) Scores, Relative to Par for Each Hole.

Participants’ median values for perceived control, task-oriented coping, irritation, tension, nervousness, and negative affect measured before each hole, and the participants’ scores on each hole are presented in Figure 1 and in Supplementary Table 1 (in which the interquartile range is included). The total mean score in relation to par across each of the 18 holes was .64 (*SD* = 1.02, *Median* = .00), giving a summed mean score in relation to par of 11.50 (*SD* = 7.10, *Median* = 12) for the entire golf round.

### Negative Affect, Task-Oriented Coping, Perceived Control, and Score

We tested nested models in which the best fitting models were the hypothesized models that contained both skill level and experience as covariates on the between person level of analysis (holes 1–18 DIC = 6,181.90; holes 1–6 DIC = 1,872.65; holes 7–12 DIC = 2,547.84; holes 13–18 DIC = 2,288.72). Model fit values for all tested models are presented in Table 1. Complete results for all tested relations for *within-person* statistical effects for whole competition rounds are presented in Table 2, and complete results for *between-person* associations are available in the Supplementary Table 2.

**Table 1.** Deviance Information Criteria (DIC) for Tested Models.

Holes	All cov	HCP	Experience	No cov
Models 1–4				
1–18	6,181.90	6,222.32	9,267.32	12,029.31
1–6	1,872.65	2,558.85	2,655.11	3,507.29
7–12	2,547.84	3,193.08	3,334.89	3,342.21
13–18	2,288.72	2,330.21	3,157.78	3,186.19
Models 5–8				
1–18	13,923.24	16,648.50	16,958.78	17,046.74
1–6	4,164.59	4,881.32	4,964.41	5,745.88
7–12	4,798.48	5,668.71	5,727.36	6,552.45
13–18	4,884.77	5,565.36	5,700.78	6,197.64

*Note.* All cov = models including all covariates; HCP = models only including golf handicap; Experience = models including only experience; No cov = models including no covariates.

**Table 2.** Standardized Within Person Effects With Combined Affect Score.

	1–18		1–6		7–12		13–18	
	β (SD)	95% CI	β (SD)	95% CI	β (SD)	95% CI	β (SD)	95% CI
Score → score	–.03 (.05)	[–.13, 05]	–.07 (.08)	[–.23, .09]	–.21 (.08)	[–.37, –.05]*	–.03 (.08)	[–.17, .11]
Cope →cope	.36 (.06)	[.26, .47]*	.08 (.09)	[–.10, .25]	.27 (.08)	[.10, .43]*	–.04 (.07)	[–.17, .08]
Cont → cont	.31 (.05)	[.20, .40]*	.06 (.09)	[–.11, .22]	.03 (.08)	[–.13, .19]	.16 (.07)	[.00, .29]*
Aff → Aff	.47 (.04)	[.38, .55]*	.29 (.09)	[.11, .45]*	.22 (.08)	[.07, .36]*	.08 (.09)	[–.11, .26]
Cope → score	.05 (.07)	[–.09, .17]	.11 (.10)	[–.09, .28]	–.01 (.11)	[–.21, .20]	.01 (.11)	[–.21, .20]
Cont → score	.02 (.06)	[–.12, .14]	–.03 (.10)	[–.23, .17]	.03 (.09)	[–.14, .22]	.00 (.11)	[–.22, .23]
Aff → score	–.05 (.06)	[–.14, .09]	.08 (.09)	[–.10, .26]	–.28 (.09)	[–.43, –.10]*	–.04 (.09)	[–.24, .13]
Score → cope	–.29 (.04)	[–.37, –.20]*	–.14 (.09)	[–.31, .03]	–.38 (.07)	[–.52, –.22]	–.25 (.09)	[–.39, –.09]*
Score → cont	–.31 (.04)	[–0.39, –.24]*	–.20 (.09)	[–.36, –.01]*	–.25 (.07)	[–.38, –.11]*	–.36 (.07)	[–.48, –.20]*
Score → aff	.33 (.04)	[.25, 41]*	.26 (.08)	[.10, .44]*	.36 (.08)	[.20, .51]*	.31 (.08)	[.15, .44]*

*Note.* → = the direction of the prediction; Score = score on golf holes; Cope = task oriented coping; Cont = perceived control; Aff = negative affect.

*A statistically credible effect where the credibility interval does not pass thru zero.

#### Within Person Relationships

In this section we report only those relationships presented in Table 2 that were statistically credible variable by variable (and not model for model), starting with a specification of the full model (i.e., containing relationships for the whole round) and then followed by specifications of models based on beginning, middle, and end of the round. Those relationships that included a zero contain a latter decimal in the direction of the credible result presented. For *score*, only the middle third had a statistically significant relationship, a negative auto regression (*β* = –.21 CI = –.37, –.05), indicating strong effects between the same variable over time.

*Perceived control* showed a positive autoregressive effect for holes 1-18 (All β = 31, CI = .20, .40) and for the last six holes (*β* = 16, CI = 00, .29). Score negatively predicted perceived control for holes 1-18 (All β = –.31, CI = –.39, –.24), and for each of the three sections of the round (holes 1-6 *β* = –.20, CI = –.36, –.01; holes 7-12 *β* = –.25 CI = –.38, –.11; and holes 13-18 *β* = –.36, CI = –.48, –.20).

*Task-oriented coping* showed a positive autoregressive effect overall (*β* = .36, CI = .26, .47), and for the middle third of the round (*β* = .27, CI = .10, .43). Score influenced the perception of ability to cope on the following hole overall (*β* = –.29, CI = –.37, –.20), and for the last two thirds of the round (holes 7-12 β = –.38, CI = –.52, –.22; and holes 13-18 *β* = –.25, CI = –.39, –.09).

*Negative affect* had positive auto-regression for all holes (All *β* = .47, CI = .38, .55), and for the first two thirds of the round (holes 1-6 *β* = .29, CI = .11, .45; and holes 7-12 *β* = .22, CI = –.07, .36). Negative affect also negatively predicted the consecutive score for the middle third of the round (*β* = –.28, CI = –.43, –.10). In the opposite relationship, higher scores positively predicted higher negative affect overall (*β* = .33, CI = .25, .41) and across the three segments of the round (holes 1-6 β = .26, CI = .10, .44; holes 7-12 β = .36 CI = .20, .51; and holes 13-18 β = .31, CI = .15, .44).

#### Between-Person Relations

All between-person relationships are reported in the Supplementary Table 2. In this section only those relations that were statistically credible are reported. Overall, there was a high degree of variation between the participants. Only one between-person relationship was statistically credible over all 18 holes, and this was the relationship between the golfers’ skill levels and their scores; a higher handicap (lower skill) predicted a higher score (All *β* = .79, CI = .35, .95). The same relationship was shown for the last third of the rounds (All *β* = .74, CI = .39, .93). Individuals with a higher handicap had higher autoregression for score during the last third of the rounds, meaning that a poorly scored hole was more often followed by additional poorly scored holes for less skilled players (*β* = .55, CI = .07, .83).

### Irritability, Nervousness, Tension, Task-Oriented Coping, Perceived Control, and Score

We tested nested models in which the best fitting models were the hypothesized models containing both skill level and experience as covariates on the between person level (holes 1-18 DIC = 13,923.24; holes 1-6 DIC = 4,164.59; holes 7-12 DIC = 4,798.48; and holes 13-18 DIC = 4,884.77). Model fit values for all tested models are presented in Table 1. Complete results for all tested relationships for within-person statistical effects for the entire competition rounds are presented in Table 3, and complete results for between-person associations are available in Supplementary Table 3.

**Table 3.** Standardized Within Person Effects Where Emotions Are Separated.

	ALL		1–6		7–12		13–18	
	β (SD)	95% CI	β (SD)	95% CI	β (SD)	95% CI	β (SD)	95% CI
Score → score	.08 (.05)	[–.18, .01]	–.14 (.08)	[–.29, .02]	–.22 (.08)	[–41, –.09]*	–.05 (.07)	[–.18, .10]
Cope →cope	.35 (.05)	[.24, .44]*	.07 (.09)	[–.10, .26]	.25 (.08)	[.08, .39]*	–.02 (.08)	[–.17, .13]
Cont → cont	.27 (.06)	[.15, .38]*	.04 (.09)	[–.12, .22]	.04 (.09)	[–.11, .20]	.16 (.07)	[.01, .30]*
Irr → irr	.31 (.06)	[.17, .40]*	.12 (.10)	[–.08, .31]	.23 (.07)	[.09, .38]*	.05 (.09)	[–.13, .22]
Nerv → nerv	.51 (.04)	[.41, .59]*	.18 (.09)	[.00, .35]*	.11 (.08)	[–.05, .26]	.01 (.10)	[–.18, .20]
Tens → tens	.36 (.05)	[.27, 46]*	.10 (.08)	[–.06, .27]	–.05 (.07)	[–.17, .11]	.02 (.09)	[–.15, .19]
Cope → score	–.01 (.07)	[–.15, .11]	.07 (.09)	[–.11, .24]	–.06 (.09)	[–.22, .14]	–.02 (.10)	[–.21, .18]
Cont → score	.01 (.07)	[–.15, 14]	–.03 (.10)	[–.21, .18]	.02 (.09)	[–.18, .18]	.01 (.11)	[–.21, .20]
Irr → score	–.13 (.06)	[–24, –.03]*	–.07 (.09)	[–.23, .11]	–.20 (.09)	[–.37, –.01]*	–.13 (.109	[–.32, .05]
Nervous → score	–.02 (.07)	[–.17, .11]	.04 (.12)	[–.21, .23]	–.02 (.08)	[–.20, .16]	–.07 (.10)	[–.26, .12]
Tens → score	.03 (.08)	[–.15, .20]	.17 (.11)	[–.04, .38]	–.12 (.09)	[–.28, .04]	.09 (.10)	[–.12, .29]
Score → cope	–.30 (.05)	[–.41, –.21]*	–.16 (.09)	[–.33, .01]	–.38 (.07)	[–.50, –.23]*	–.23 (.08)	[–.38, –.06]*
Score → cont	–.33 (.04)	[–.41, –.25]*	–.16 (.09)	[–.32, .03]	–.26 (.08)	[–.42, –.09]*	–.34 (.07)	[–.48, –.19]*
Score → irr	.43 (.04)	[.41, .59]*	.25 (.08)	[.09, .38]*	.53 (.07)	[.37, .62]*	.41 (.07)	[.27, .54]*
Score → nerv	–02 (.04)	[–.10, .06]	.09 (.10)	[–.10, .27]	–.04 (.09)	[–.22, .15]	–.08 (.08)	[–.24, .08]
Score → tens	.10 (.05)	[.01, .21]*	.16 (.10)	[–.06, .35]	.12 (.10)	[–04, .31]	.10 (.09)	[–.06, .27]

*Note.* → = the direction of the prediction; Score = score on golf holes; Cope = task oriented coping; Cont = perceived control; Irr = irritability; Nerv = nervousness; tens = tension.

*A statistically credible effect where the credibility interval does not pass thru zero.

#### Within-Person Relations

Of all the relationships shown in Table 3, in this section we report only those relationships that were statistically credible. As in the model with the latent affect variable, *score* had a negative autoregressive effect in the middle third of the round (holes 7-12 *β* = –.22 CI = –.41, –.09).

For *perceived control* there was a positive autoregressive effect overall (*β* = .27, CI = .15, .38) and for the last six holes (*β* = 16, CI = .01, .30). Score negatively predicted perceived control overall (*β* = –.33, CI = –.41, –.25) and during the last two thirds of the round (Holes 7-12 *β* = –.26 CI = –.42, –.09; and holes 13-18 *β* = –.34, CI = –.48, –.19).

*Task-oriented coping* showed a positive autoregressive effect overall (*β* = .35, CI = .24, .44) and during the middle of the round (*β* = .25, CI = .08, .39). Score negatively predicted the ability to cope with the subsequent hole both overall (*β* = –.30, CI = –.41, –.21) and for the last two thirds of the round (holes 7-12 *β* = –.38, CI = –.50, –.23; and holes 13-18 *β* = –.23, CI = –.38, –.06).

*Irritability* showed positive autoregressive effects overall (*β* = .31, CI = .17, .40) and in the middle third of the round (*β* = .23, CI = .09, .38). There was a negative relation between irritation and score in the overall round (*β* = –.13, CI = –.24, –.03) and for the middle third of the round (*β* = –.20, CI = –.37, –.01). Score positively predicted irritability overall (*β* = .43, CI = .41, .59) and for each third of the round (holes 1-6 *β* = .25, CI = .09, .38; holes 7-12 *β* = .53, CI = .37, .62; and holes 13-18 *β* = .41, CI = .27, .54).

*Nervousness* had a positive auto-regression overall (*β* = .51, CI = .41, .59) and for the first third of the round (*β* = 18, CI = .00, .35) but for no other tested relationship.

Perceived *tension* showed a positive auto-regression overall (*β* = .36, CI = .27, .46) and for the last third of the golf round (*β* = .02, CI = –.15, .19). Score predicted perceived tension overall (*β* = .33, CI = .25, .41).

#### Between-Person Relations

All between-person relations are reported in the Appendix, Table A2; and, in this section, only those relations that were statistically credible are reported. As for model 1, the variation between golfers was very high overall. Two between-person relationships were statistically credible over all 18 holes: (a) the relation between skill level and score where higher handicap (lower skill) predicted a higher score (*β* = .71, CI = .32, .89), and (b) the same relationship in the last third of the rounds (*β* = .63, CI = .09, .89). Lower skill level was related to the auto-regression for score (i.e., less skilled players more often followed a bad hole with another bad hole; *β* = .54, CI = .00, .84). During the middle third of the round, more skilled players also reported less irritation after a poorly scored hole than did better players (*β* = –.67, CI = –.90, –.03), but they showed a stronger relation between score and tension on the following hole (*β* = .74, CI = .02, .96).

## Discussion

The present study examined whether golfers’ negative affect, perceived control, and task-oriented coping measured between golf holes predicted their performance on subsequent holes across an 18-hole competition. Additionally, we assessed whether performance on the preceding hole predicted negative affect, perceived control, and task-oriented coping on the ensuing hole. The within-person relationships of various negative affect measures were largely in line with previous research from other performance domains. They showed that golfers’ performance-induced affects (i.e., valence, dominance and arousal) were stronger than their emotion-induced performances (Schantz & Conroy, 2009). One particularly interesting finding was that, during certain temporal aspects of the competition round (i.e., holes 7-12), negative affect predicted a better score on the subsequent hole. Perhaps unsurprisingly, between-person relationships revealed that more skilled golfers performed better than less skilled players and were less influenced by bad performances, both with regard to the subsequent score and their affective response following a bad score. Poor scores also predicted lower perceptions of control and task-oriented coping in relation to the upcoming hole. These observed effects of negative affect and negative discrete emotions are in line with previous studies both in sport and school environments (Doron & Gaudreau, 2014; Hanton & Connaughton, 2001; Schantz & Conroy, 2009; Skinner et al., 1998).

Interestingly, our more exploratory analysis showed that separating a total score of negative affect into separate discrete emotions (i.e., irritation, tension, and nervousness) offered more precise insight into the differential effects of athletes’ emotional states. The most striking result in this study was that golfers with high levels of irritability performed better on the subsequent hole. This association was statistically credible for the whole round, but when the round was divided into thirds it was only statistically credible for the middle of the round (i.e., holes 7-12). There is some limited evidence that, for some athletes, feelings of anger in the time period before a competition can predict better golf performance (Hassmén et al., 1998). The finding that irritability and negative affect were associated with improved subsequent performance may be partially supported by theories outlining emotion regulation in sport (Lane et al., 2012). An alternative explanation may be a regression towards the mean (Connolly & Rendleman, 2008; Galton, 1886), such that in the present study an extremely poor performance tended to be followed by a less extreme (i.e., on par) performance. The observed effect between performance score and subsequent irritation demonstrated that golfers experience a negative affective response to poorly performed holes. Further, within the middle third of the round there was a negative auto regression for score suggesting that a bad hole was typically followed by a better hole. There is the distinct possibility that the negative relation between irritation and the score on the following hole was actually due to the fact that the poor performance on the previous hole, rather than the irritation associated with it, predicted the better subsequent performance. On the whole, it is not likely that becoming more irritated, nervous, and tense directly promoted better performance. Further research is needed in order to investigate this relationship in more controlled settings.

The within-person relation in which a poor score was followed by less perceived control and task-oriented coping is in line with previous studies with similar point-by-point research designs (Doron & Gaudreau, 2014; Schantz & Conroy, 2009). One way to interpret this could be that the relations between perceived control and task-oriented coping could be perceived as connected to confidence (Hanton & Connaughton, 2002) which would indicate a bad spiral of negative results, predicting even less perceived control and task-oriented coping. However, since neither perceived control nor coping showed any opposite relation to score, and the autoregression of score tended to be negative (a poor score was followed by a better score), we do not have evidence to support that proposition. Future studies may aim to evaluate the influence of perceived control by either manipulating it as an independent variable or through the use of more detailed within person analyses. In hindsight, using both task-oriented coping and perceived control might not have added much information.

Between-person analyses uncovered very large individual differences among golfers, meaning that relatively high effect sizes were not statistically credible. This suggests that the variable centered approach in this study might not offer a complete picture of the relationships between negative affect, emotions, perceived control, task-oriented coping, and subsequent performance. It may be interesting to explore the characteristics of participant subgroups (e.g., personality) who display differing affective responses to their performance and/or differing performances in relation to their affective states. In support of this exploration, some research has shown that personality can play a role in the relationship between pre-competitive mood and subsequent performance (Hassmén et al., 1998) as well as having a direct impact on performance in pressure situations (Geukes et al., 2017; Graydon & Murphy, 1995; Roberts et al., 2013). Therefore, a paucity of measured personality moderators in this study is a limitation and should be acknowledged in future research.

## Limitations and Directions for Future Research

While the present study was novel in its approach to investigating the relationships between negative affective states, emotions, task-oriented coping, and performance via repeated measures collected during a golf competition and offered new insights into the complex interrelationships of these important variables, there were study limitations that should be addressed in further research. First, although measuring emotions between each hole provided a new perspective, more precise data might be obtained by collecting stroke-by-stroke data. Admittedly, this sampling frequency might be so intrusive as to induce interfering observation effects, especially when measuring multiple variables. In fact, it is possible that even hole-by-hole reporting was intrusive in this way, leading two of our participants to withdraw from the study. Future investigators might collect inobtrusive physiological data such as heart rate (Balk et al., 2013) or heart rate variability, skin conductance and temperature as proxies for psychological arousal and stress, and might measure performance via golf technology (e.g., GPS trackers), all within actual golf competition. Second, this study primarily focused on negative affect, and we did not specifically measure stress, arousal, and anxiety. Broadening the measurement of emotions might better capture the complexity of golfers’ emotional states during competition. Third, while collecting data during competition provided greater ecological validity, our limited control over test variables restricted our ability to make conclusive determinations of cause and effect relationships between emotions and performance. Additional experimental research designs, like those that have tested several aspects of putting performance (Balk et al., 2013; Gucciardi & Dimmock, 2008), might offer further insight regarding variables of interest in the present study. Validated biological measurement techniques (e.g., heart rate, eye tracking) that can be integrated within golf simulator performance data might permit better research control over competition conditions, perceived pressures, and performance outcomes (Woodman & Davis, 2008). Fourth, our study design does not permit us to rule out moderator influences on these variables. In our study and others, more skilled golfers seemed to perform better under pressure, compared to less skilled golfers (Balk et al., 2013; Gucciardi & Dimmock, 2008). However, we could not determine whether a higher skill level may have created less frustration, a greater potential to recover from bad holes, or whether better players also have better emotional self-regulation. Therefore, the causal effects of these associations need further investigation.

## Conclusion

The main finding from this study was that neither emotions nor perceived ability to control or handle the game, appeared to be of central importance in predicting players’ subsequent performance during a golf competition. Skilled players appeared to be more effective in managing their responses to bad holes compared with less skilled players, in terms of both their affective responses and their performance on subsequent holes. Practical implications for competitive golfers and their coaches, were that skill level (i.e., HCP) appeared to directly impact both performance scores and the relationship between negative affect and scores. It is possible that better players are not only more skilled technically (e.g., mechanics of swing), but they are also better at the cognitive elements of the game, such as re-focusing after a bad shot and preparing themselves for the next shot, even when they are feeling frustrated and irritated. Thus, in accordance with the theoretical underpinnings of the third wave of cognitive behavioral therapy (e.g., Gardner & Moore, 2004, 2012), accepting negative emotions, such as frustration and irritation, being in the present moment and focusing on task relevant cues would seem to be effective ways of optimizing performance. Until there is a clearer indication of causal directions between these associations, coaches and sport psychologists may be well advised to help players improve their performance by addressing both their playing skill and their use of acceptance-based psychological interventions in learning to focus on the next shot, independent of which emotions may be perceived at the moment.

## Supplemental Material

sj-pdf-1-pms-10.1177_00315125211005938 - Supplemental material for Relating Competitive Golfers’ Perceived Emotions and Performance Click here for additional data file. Supplemental material, sj-pdf-1-pms-10.1177_00315125211005938 for Relating Competitive Golfers’ Perceived Emotions and Performance by Erik Lundkvist, Henrik Gustafsson, Gunilla Björklund, Paul Davis and Andreas Ivarsson in Perceptual and Motor Skills

sj-pdf-2-pms-10.1177_00315125211005938 - Supplemental material for Relating Competitive Golfers’ Perceived Emotions and Performance Click here for additional data file. Supplemental material, sj-pdf-2-pms-10.1177_00315125211005938 for Relating Competitive Golfers’ Perceived Emotions and Performance by Erik Lundkvist, Henrik Gustafsson, Gunilla Björklund, Paul Davis and Andreas Ivarsson in Perceptual and Motor Skills
