# Supplementary material for: Relating Competitive Golfers’ Perceived Emotions and Performance
Source: Percept Mot Skills. 2021 Apr 12;128(4):1549–68. doi: 10.1177/00315125211005938 (PMC8267075; doi:10.1177/00315125211005938)
Supplement: sj-pdf-1-pms-10.1177_00315125211005938 - Supplemental material for Relating Competitive Golfers’ Perceived Emotions and Performance [file sj-pdf-1-pms-10.1177_00315125211005938.pdf]

Supplementary table 1

Descriptive statistics. Median and interquartile range

	Score	Negative affect	Control	Coping	Irritability	Nervousness	Tension
Hole 1	.00 (.00-1.00)	3.67 (2.62-4.68)	7.20 (6.08-8.43)	7.00 (5.43-8.34)	1.10 (.50-2.75)	5.10 (2.68-6.55)	4.10 (2.00-6.30)
Hole 2	1.00 (.00-1.00)	2.80 (1.53-4.13)	7.10 (5.90-8.35)	7.00 (5.95-8.35)	1.50 (.70-3.15)	3.30 (1.35-5.85)	2.80 (1.65-4.25)
Hole 3	.00 (.00-1.00)	2.97 (1.48-4.28)	7.00 (5.70-7.90)	6.95 (5.70-8.00)	2.35 (.90-3.95)	2.80 (1.45-5.10)	2.90 (1.33-4.88)
Hole 4	.50 (.00-1.00)	2.95 (1.47-3.79)	6.60 (5.18-7.93)	6.40 (5.45-7.85)	1.30 (1.08-3.10)	3.05 (1.20-4.40)	2.95 (1.00-4.55)
Hole 5	.00 (.00-1.00)	2.27 (1.28-3.55)	5.95 (4.33-7.83)	6.00 (4.80-7.05)	2.80 (1.05-4.35)	1.70 (1.05-3.55)	2.00 (1.05-3.65)
Hole 6	.00 (.00-1.00)	2.87 (2.00-3.78)	6.10 (4.53-7.13)	6.15 (4.70-7.60)	3.00 (1.18-4.60)	2.0 0 (1.18-3.98)	2.30 (1.25-3.83)
Hole 7	1.00 (.00-1.00)	2.87 (1.82-4.23)	5.90 (5.25-7.70)	6.20 (4.08-8.10)	2.15 (1.13-4.40)	2.50 (1.18-5.03)	2.85 (1.68-4.83)
Hole 8	.00 (.00-1.00)	2.78 (1.61-3.57)	6.30 (4.78-7.43)	6.15 (4.09-7.53)	3.00 (1.78-5.13)	1.55 (1.0-3.70)	2.05 (1.18-3.70)
Hole 9	.00 (.00-1.00)	1.93 (1.52-3.17)	6.10 (4.70-7.70)	6.50 (4.10-8.30)	2.20 (1.05-3.80)	1.30 (.60-2.50)	2.10 (.90-2.95)
Hole 10	1.00 (.00-2.00)	2.23 (1.02-2.88)	6.05 (3.85-7.30)	6.50 (4.11-8.05)	1.95 (.68-3.83	1.30 (.60-2.93)	1.65 (.88-3.10)
Hole 11	.00 (.00-1.00)	2.62 (.98-3.61)	5.40 (3.50-7.70)	6.05 (4.12-8.00)	2.20 (1.00-4.70)	1.45 (.90-3.18)	1.65 (.95-4.60)
Hole 12	1.00 (.00-1.00)	1.80 (.99-3.43)	6.25 (4.30-8.10)	6.50 (4.13-7.93)	2.15 (.98-4.05)	1.55 (.78-3.33)	1.30 (.85-2.53)
Hole 13	.00 (.00-1.00)	2.70 (1.32-3.35)	6.10 (3.80-7.70)	6.10 (4.14-7.80)	3.40 (1.35-6.10)	1.10 (.65-2.90)	1.90 (.95-4.10)
Hole 14	.00 (.00-1.00)	1.83 (.90-3.69)	6.40 (3.90-8.05)	6.55 (4.15-8.65)	1.50 (.88-4.55)	1.25 (.85-3.53)	1.75 (.82-3.65)
Hole 15	.00 (.00-1.25)	1.87 (1.33-3.33)	6.15 (4.03-7.05)	6.40 (4.16-7.93)	2.30 (1.08-4.48)	1.40 (.75-3.73)	2.00 (.75-3.35)
Hole 16	.00 (.00-1.00)	3.13 (1.00-3.98)	5.85 (3.13-7.50)	5.20 (4.17-7.95)	2.80 (1.15-5).28	1.20 (.75-3.85)	1.85 (.78-4.13)
Hole 17	1.00 (.00-1.00)	1.90 (.86-3.62)	6.25 (3.58-8.30)	6.40 (4.18-8.35)	1.65 (.73-5).40)	1.10 (.80-2.95)	2.00 (1.00-3.78)
Hole 18	.00 (.00-1.00)	2.30 (.83-4.07)	5.90 (3.25-7.95)	6.60 (4.19-8.05)	2.00 (.85-5.90)	1.20 (.65-3.30)	1.40 (.85-4.25)

Supplementary table 2.

Between person associations including score, coping, control and negative affect

		1-18		1-6		7-12		13-18	
		β (SD)	95% CI	β (SD)	95% CI	β (SD)	95% CI	β (SD)	95% CI
Skill → score		.79 (.15)	[.35, .95]*	.43 (.28)	[-.19, .85]	.46 (.25)	[-.16, .84]	.74 (.16)	[.39, .93]*
Experience → score		.00 (.19)	[-.38, .34]	-.39 (.29)	[-.84, .21]	.02 (.28)	[-.59, .50]	.27 (.23)	[-.29, .61]
Skill → coping		-.07 (.21)	[-.47, .32]	-.25 (.21)	[-.64, .19]	.02 (.22)	[-.36, .43]	.03 (.21)	[-.43, .38]
Experience → coping		.35 (.21)	[-.15, .64]	.14 (.21)	[-.32, .53]	.32 (.20)	[-.12, .64]	.41 (.21)	[-.03, .70]
Skill → control		-.09 (.21)	[-.46, .30]	-.32 (.21)	[-.66, .14]	-.04 (.22)	[-.40, .39]	.03 (.22)	[-.45, .38]
Experience → control		.29 (.21)	[-.15, .63]	.05 (.22)	[-.36, .45]	.32 (.21)	[-.11, .66]	.39 (.20)	[-.04, .69]
Skill → negative affect		-.10 (.19)	[-.44, .26]	.18 (.23)	[-.26, .59]	-.08 (.23)	[-.49, .40]	.28 (.19)	[-.62, .15]
Experience → negative affect		-.36 (.20)	[-.64, .09]	-.10 (.22)	[-.49, 35]	-.28 (.22)	[-.64, .21]	-.38 (.21)	[-.68, .13]
Autoreg. score	Skill	.48 (.29)	[-.40, .85]	.48 (.34)	[-.37, .96]	-.50 (.43)	[-.97, .44]	.55 (.22)	[.07, .83]
	Experience	.54 (.23)	[-.14, .85]	.54 (.35)	[-.42, .94]	.28 (.35)	[-.35, .88]	.64 (.24)	[-.05, .93]
Autoreg. coping	Skill	-.25 (.31)	[-.76, .43]	.31 (.39)	[-.56, .84]	-.60 (.36)	[-.97, .37]	.37 (.38)	[-.52, .84]
	Experience	-.02 (.28)	[-.55, .53]	.17 (.46)	[-.78, .85]	-.21 (.31)	[-.74, .45]	.01 (.54)	[-.90, .87]
Autoreg. control	Skill	.05 (.25)	[-.52, .45]	.60 (.26)	[-.12, .90]	-.50 (.28)	[-.89, .20]	.12 (.32)	[-.56, .66]
	Experience	.16 (.27)	[-.36, .68]	.52 (.28)	[-.20, .87]	-.43 (.31)	[-.87, .34]	.07 (.36)	[-.64, .67]
Autoreg. Negative affect	Skill	-.11 (.26)	[-.57, .38]	-.04 (.45)	[-.78, .85]	.43 (.36)	[-.38, .92]	-.29 (.33)	[-.77, .56]
	Experience	-.07 (.26)	[-.64, .36]	.03 (.44)	[-.88, .77]	.01 (.35)	[-.71, .62]	-.33 (.42)	[-.86, .57]
Coping → score	Skill	.01 (.54)	[-.97, .84]	-.01 (.44)	[-.83, .76]	.19 (.47)	[-.83, .85]	.14 (.42)	[-.64, .84]
	experience	.24 (.42)	[-.58, .91]	-.19 (.48)	[-.88, .77]	.38 (.38)	[-.53, .89]	.25 (.47)	[-.89, .84]
Cont → score	Skill	-.33 (.36)	[-.91, .35]	-.05 (.43)	[-.71, .90]	-.27 (.45)	[-.86, .77]	-.46 (.39)	[-.91, .48]
	experience	-.31 (.45)	[-.89, .92]	-.38 (.41)	[-.93, .58]	-.28 (.44)	[-.92, .69]	-.24 (.45)	[-.90, .71]
Irritability → score	Skill	-.34 (.34)	[-.88, .46]	-.67 (.30)	[-.95, .25]	-.46 (.43)	[-.94, .49]	-.39 (.37)	[-.79, .58]
	Experience	-.51 (.32)	[-.90, .41]	-.30 (.37)	[-.82, .72]	.20 (.46)	[-.64, .93]	-.21 (.37)	[-.81, .59]
Nervousness → score	Skill	-.10 (.29)	[-.63, .53]	-.42 (.31)	[-.85, .34]	.34 (.29)	[-.32, .75]	-.33 (.41)	[-.93, .66]
	Experience	-.05 (.26)	[-.58, .38]	-.07 (.34)	[-.57, .66]	-.01 (.30)	[-.66, .47]	.10 (.43)	[-.68, .85]

Tension → score	Skill	.06 (.22)	[-.39, .43]	-.62 (.36)	[-.95, .43]	.07 (.30)	[-.56, .59]	.08 (.32)	[-63, .56]
	Experience	.30 (.21)	[-.30, .58]	-.14 (.36)	[-.75, .63]	.14 (.27)	[-.39, .66]	.31 (.32)	[-.42, .77]
Score → coping	Skill	.52 (.24)	[-.15, .87]	-.04 (.30)	[-.63, .53]	.52 (.35)	[-.31, .93]	.66 (.25)	[.07, .95]*
	Experience	.30 (.21)	[-.23, .62]	.30 (.31)	[-.34, .84]	.06 (.31)	[-.60, .52]	.28 (.37)	[-.68, .72]

Note. * A statistically credible effect where the credibility interval does not pass thru zero.

Supplementary table 3.

Between person associations including score, coping, control, irritability, nervousness and tension

		1-18		1-6		7-12		13-18	
		β (SD)	95% CI	β (SD)	95% CI	β (SD)	95% CI	β (SD)	95% CI
Skill → score		.71 (.16)	[.32, .89]*	.35 (.24)	[-.14, .77]	.37 (.26)	[-.32, .77]	.63 (.21)	[.09, .89]*
Experience → score		-.03 (.23)	[-.48, .43]	-.30 (.28)	[-.76, .28]	-.05 (.28)	[-.55, .49]	.21 (.24)	[-.35, .60]
Skill → coping		.01 (.23)	[-.48, .34]	-.21 (.22)	[-.59, .25]	.03 (.21)	[-.39, .44]	.04 (.21)	[-.40, .41]
Experience → coping		.40 (.21)	[-.15, .67]	.13 (.21)	[-.30, .52]	.30 (.19)	[-.08, .62]	.37 (.20)	[-.07, .68]
Skill → control		-.04 (.23)	[-.51, .34]	-.26 (.21)	[-.62, .22]	-.01 (.21)	[-.39, .44]	.04 (.21)	[-.41, .41]
Experience → control		.32 (.21)	[-.16, .62]	.04 (.22)	[-.32, .45]	.30 (.20)	[-.09, .65]	.36 (.20)	[-.10, .66]
Skill → irritation		-.19 (.21)	[-.51, .32]	-.14 (.24)	[-.52, .38]	-.25 (.20)	[-.58, .21]	-.03 (.22)	[-.45, .41]
Experience → irritation		-.24 (.22)	[-.56, .19]	-.08 (.25)	[-.52, .45]	-.19 (.20)	[-.53, .26]	-.22 (.22)	[-.57, .23]
Skill → nervous		.09 (.23)	[-.45, .44]	.34 (.21)	[-.13, .65]	.15 (.19)	[-.28, .48]	-.29 (.20)	[-.62, .17]
experience → nervous		-.26 (.24)	[-.62, .27]	.03 (.22)	[-.39, .42]	-.20 (.22)	[-.56, .22]	-.30 (.20)	[-.59, .17]
Skill → tension		-.05 (.21)	[-.49, .33]	.16 (.22)	[-.31, .52]	-.02 (.22)	[-.45, .36]	-.31 (.20)	[-.62, .16]
Experience → tension		-.26 (.23)	[-.62, .33]	.02 (.22)	[-.41, .46]	-.21 (.22)	[-.60, .31]	-.31 (.20)	[-.61, .19]
Autoreg. score	Skill	.54 (.23)	[.00, .84]*	.31 (.40)	[-.61, .83]	-.66 (.39)	[-.94, .55]	.55 (.24)	[-.11, .85]
	Experience	.60 (.24)	[-.01, .88]	.43 (.42)	[-.73, .91]	-.35 (.35)	[-.76, .40]	.61 (.24)	[-.06, .89]
Autoreg. coping	Skill	-.12 (.27)	[-.62, .47]	.17 (.40)	[-.67, .84]	-.61 (.34)	[-.96, .31]	.28 (.39)	[-.61, .84]
	Experience	.02 (.28)	[-.49, .53]	-.22 (.47)	[-.96, .78]	-.27 (.30)	[-.72, .36]	-.02 (.49)	[-.89, .86]
Autoreg. control	Skill	-.02 (.27)	[-.50, .49]	.57 (.30)	[-.19, .92]	-.44 (.26)	[-.87, .13]	.06 (.33)	[-.57, .68]
	Experience	.12 (.27)	[-.41, .56]	.57 (.28)	[-.18, .91]	-.56 (.30)	[-.91, .15]	.03 (.39)	[-.72, .72]
Autoreg. Irritability	Skill	-.10 (.25)	[-.62, .38]	-.10 (.38)	[-.75, .62]	.02 (.41)	[-.63, .89]	.09 (.30)	[-.50, .60]
	Experience	.06 (.27)	[-.43, .60]	.32 (.40)	[-.54, .88]	-.07 (.35)	[-.72, .56]	.05 (.38)	[-.68, .72]
Autoreg. Nervousness	Skill	.12 (.32)	[-.54, .71]	.26 (.36)	[-.56, .76]	-.32 (.39)	[-.88, .76]	-.06 (.38)	[-.73, .67]
	Experience	-.04 (.28)	[-.58, .50]	.06 (.35)	[-.68, .66]	-.30 (.43)	[-.93, .72]	-.21 (.47)	[-.87, .76]
	Skill	.19 (.27)	[-.42, .65]	.05 (.47)	[-.76, .87]	.28 (.45)	[-.69, .86]	.41 (.31)	[-.31, .83]

Autoreg. tension	Experience	-.14 (.30)	[-.62, .58]	-.19 (.43)	[-.88, .69]	-.25 (.42)	[-.92, .57]	-.03 (.37)	[-.77, .60]
Coping → score	Skill	.27 (.43)	[-.79, .91]	.14 (.42)	[-.72, .83]	.25 (.43)	[-.60, .89]	.27 (.46)	[-.84, .88]
	experience	.38 (.44)	[-.64, .98]	-.20 (.47)	[-.87, .75]	-.39 (.34)	[-.89, .35]	.27 (.44)	[-.70, .90]
Cont → score	Skill	-.46 (.38)	[-.84, .61]	.21 (.45)	[-.67, .89]	-.09 (.39)	[-.73, .68]	-.07 (.52)	[-.91, .90]
	experience	-.35 (.39)	[-.82, .66]	-.20 (.43)	[-.87, .60]	-.44 (.44)	[-.94, .61]	-.35 (.44)	[-.94, .67]
Irritability → score	Skill	-.36 (.42)	[-.77, .75]	.18 (.39)	[-.62, .82]	-.49 (.23)	[-.82, .15]	-.26 (.40)	[-.85, .55]
	Experience	-.40 (.48)	[-.90, .80]	-.22 (.45)	[-.91, .68]	-.67 (.23)	[-.90, -.03]*	.19 (.49)	[-.72, .93]
Nervousness → score	Skill	-.50 (.23)	[-.86, .11]	-.76 (.31)	[-.98, .12]	-.59 (.36)	[-.94, .41]	-.42 (.32)	[-.85, .41]
	Experience	-.48 (.26)	[-.81, .19]	.17 (.38)	[-.49, .90]	-.01 (.36)	[-.71, .67]	-.58 (.35)	[-.93, .41]
Tension → score	Skill	.50 (.30)	[-.36, .90]	-.46 (.42)	[-.90, .77]	.21 (.32)	[-.47, .74]	.58 (.29)	[-.18, .92]
	Experience	.56 (.43)	[-.52, .90]	-.47 (.40)	[-.93, .57]	.74 (.24)	[.02, .96]*	.15 (.30)	[-.51, .63]
Score → coping	Skill	-.12 (.30)	[-.61, .37]	-.28 (.33)	[-.85, .42]	.36 (.30)	[-.41, .74]	-.39 (.42)	[-.93, .58]
	Experience	.02 (.28)	[-.56, .51]	.02 (.35)	[-.55, .75]	.06 (.30)	[-.62, .51]	.16 (.44)	[-.66, .91]
Score → control	Skill	.08 (.24)	[-.45, .51]	-.51 (.39)	[-.93, .54]	.14 (.36)	[-.73, .59]	.01 (.34)	[-.69, .59]
	Experience	.35 (.22)	[-.18, .63]	.06 (.44)	[-.76, .81]	.18 (.26)	[-.39, .62]	.34 (.33)	[-.44, .79]
Score → Irritability	Skill	.34 (.20)	[-.24, .62]	-.19 (.27)	[-.61, .45]	.10 (.39)	[-.73, .80]	.64 (.27)	[-.09, .94]
	Experience	.36 (.12)	[-.03, .71]	.00 (.27)	[-.39, .61]	.46 (.28)	[-.15, .88]	.11 (.35)	[-.72, .66]
Score → Nervousness	Skill	.34 (.29)	[-.35, .83]	.35 (.38)	[-.57, .85]	.35 (.32)	[-.23, .83]	.45 (.29)	[-.25, .85]
	Experience	-.12 (.26)	[-.66, .40]	.44 (.35)	[-.41, .87]	-.11 (.29)	[-.74, .38]	.16 (.30)	[-.47, .65]
Score → Tension	Skill	.19 (.43)	[-.86, .91]	.39 (.38)	[-.66, .86]	.32 (.41)	[-.66, .97]	.49 (.39)	[-.52, .93]
	Experience	-.22 (.32)	[-.86, .40]	.64 (.29)	[-.13, .93]	.00 (.35)	[-.71, .57]	.14 (.43)	[-.78, .78]

Note. * A statistically credible effect where the credibility interval does not pass thru zero.
